# Molecular identification and antifungal susceptibility profile of yeast from vulvovaginal candidiasis

**DOI:** 10.1186/s12879-020-04985-w

**Published:** 2020-04-19

**Authors:** Yu Shi, Yuxia Zhu, Shangrong Fan, Xiaoping Liu, Yiheng Liang, Yingying Shan

**Affiliations:** 1 Department of Obstetrics and Gynecology, Peking University Shenzhen Hospital, Shenzhen, 518036 China; 2 Shenzhen Key Laboratory of Gynecological Diagnostic Technology Research, Shenzhen, 518036 China; 3 Anhui Medical University, Hefei, 230022 China; 4 Department of Laboratory Science, Peking University Shenzhen Hospital, Shenzhen, 518036 China

**Keywords:** Candidiasis, Vulvovaginal, Candida, Identification, Antifungal susceptibility

## Abstract

**Background:**

Accurate identification Candida is important for successful therapy and epidemiology study. The aim of research is to study API 20C yeast identification system identification rate by using molecular identification as gold standard and tested the antifungal susceptibility of Candida from patients with vulvovaginal candidiasis (VVC).

**Methods:**

In total, 3574 yeast isolates were obtained from patients with VVC. API 20C yeast identification, molecular identification and in vitro antifungal susceptibility were performed.

**Results:**

*C. albicans* was the predominant Candida species [2748 isolates, 76.9%] in VVC. The isolates from vaginal samples represented 22 species based on molecular identification. The API 20C system identifies only 11 of the species encountered during the study period. Based on the API 20C system, 3273 (91.58%) isolates were correctly identified to the species level. The correct identification rate of the API 20C system for rare yeast was 15.29% (26/170 isolates). Antifungal susceptibility was tested in a total of 1844 isolates of Candida from patients with VVC. *C. albicans* was susceptible to most of the tested antifungals. The MICs of azoles for *C. glabrata* were higher than those for *C. albicans*. The MICs of echinocandins for *C. parapsilosis* were higher than those for *C. albicans*.

**Conclusions:**

The API 20C yeast identification system can be used to reliably identify the most common Candida species while molecular methods are necessary for the identification of closely related, emerging, and rare yeast species. The results from this study suggest that much of the previous studies on the epidemiology of VVC should be re-thought. *C. albicans* was susceptible to most of the tested antifungals.

## Background

An estimated 75% of women will have at least one episode of vulvovaginal candidiasis (VVC) and 40–45% will have two or more episodes [1]. The estimated probability of recurrent VVC (RVVC),which was defined as four or more episodes of symptomatic VVC within 1 year, after VVC will be 14–28% [2]. *C. albicans*, which is responsible for 85–95% of Candida vaginal infections, is the major aetiological agent involved in cases of VVC, followed by *C. glabrata* and *C. parapsilosis* [3–6]. Accurate identification at the species level is paramount for successful therapy and appropriate patient care. However, commonly used identification method, the API yeast identification system, has shown a rather inconsistent ability to identify clinical isolates with an overall identification rate ranging from 80 to 96% [7]. In addition, with the discovery of new, closely related Candida species and novel species, the correct identification of the isolates has become more difficult by using the common methods [8]. PCR and sequencing of relevant genes provide a rapid and accurate Candida speciation, genotyping of individual species, and finally, antifungal drug sensitivity [9]. Treatment options for refractory symptoms caused by antifungal resistant Candida are extremely limited. New therapeutic study, options and strategies are urgently needed to meet the challenge of drug resistance [10–15]. RVVC affects about 138 million women annually, with a global annual prevalence of 3871 per 100,000 women; 372 million women are affected by RVVC over their lifetime [11]. We reported the distribution of yeast isolates based on molecular identification from patients with VVC in southern China from 2003 to 2018 and compare the identification rate of molecular methods with API 20 C system. We also tested the common used and several potential clinical using antifungals.

## Methods

### Patients and case definition

A prospective study of 3574 consecutive patients with VVC and RVVC was conducted at the Department of Gynecology, Peking University Shenzhen Hospital from April 2003 to September 2018. The research protocol was approved by the ethics committee of the hospital, and all subjects gave their informed consent to participate. The cases of VVC in pregnancy and VVC caused by multiple Candida species were excluded. A case of VVC was defined as a patient with vulvar itching, vaginal discharge and a positive Candida culture. Confirmation was obtained by demonstration of blastoconidia and pseudohyphae on 10% potassium hydroxide preparation. Among the 3574 patients, 588 isolates (16.5%) which first found from per patient with RVVC were selected. The mean ages of patients with RVVC and VVC were 31.01 [SD 6.04] and 29.67 [SD 6.64] years, respectively (*P* < 0.05).

### Vaginal samples and API 20C identification

A sample from the lateral vaginal wall was obtained with a sterile cotton-tipped swab. The swab was placed in a tube filled with saline prior to direct microscopic examination on a wet slide, to which a drop of 10% potassium hydroxide had been added. Culture was performed on samples obtained from all cases that had positive on 10% potassium hydroxide preparation. All specimens were plated on a Sabouraud agar plate for 24–48 h at 37 °C. Isolates were identified using a standard system, API 20C [Biomerieux, France], and stored in medium containing 2% glucose, 2% peptone and 20% glycerol at − 70 °C.

### Molecular identification

Isolates were removed from the − 70 °C freezer and revived on a Sabouraud agar plate for 24–48 h at 37 °C. One single yeast colony from the isolates was suspended in a microcentrifuge tube containing 50 μL of lysis buffer for direct polymerase chain reaction (PCR) to identify fungus (Takara Biotechnology Co., Ltd., Dalian, China). The composition of the PCR mixture, and the PCR conditions were in accordance with the methods previously described [16–19]. At first, we use PCR primers *of C. albicans* complexes, *C. glabrata* complexes, and *C. parapsilosis* complexes to identify the three complexes, respectively. All other yeasts were identified by using PCR and sequencing. The primers used in this study are shown in Table 1.

**Table 1:** The primers used in this study

***Candida*** species	Primer name	Forward(5′-3′)	Reverse(5′-3′)	Amplified fragment size (bp)	References
*C. albicans* complexes	HWP1	GCTACCACTTCAGAATCATCATC	GCACCTTCAGTCGTAGAGACG	*C. albicans:839* and *941* *C. africana:700* *C. dubliniensis:569*	Shan,2014
*C. glabrata* complexes	GLA NIV BRA	CGGTTGGTGGGTGTTCTGC AGGGAGGAGTTTGTATCTTTCAAC GGGACGGTAAGTCTCCCG	ACCAGAGGGCGCAATGTG	*C. glabrata:397* *C. bracarensis*: *223* *C. nivariensis:293*	Li,2014
*C. parapsilosis* complexes	mCPF mCOF mCMF	TTTGCTTTGGTAGGCCTTCTA TAAGTCAACTGATTAACTAAT AACTGCAATCCTTTTCTTTCTA	AATATCTGCAATTCATATTACT	*C. parapsilosis:171* *C. orthopsilosis:109* *C. metapsilosis:217*	Asadzadeh,2015
Rare yeast	NL1,NL4	GCATATCAATAAGCGGAGGAAAAG-3’	GGTCCGTGTTTCAAGACGG	*500–600*	Leaw,2006

^1^Shan Y, Fan S, Liu X, et al. Prevalence of *Candida albicans*-closely related yeasts, *Candida africana* and *Candida dubliniensis*, in vulvovaginal candidiasis. Med Mycol, 2014, 52 (6): 636–40.

^2^Li J, Shan Y, Fan S, et al. Prevalence of *Candida nivariensis* and *Candida bracarensis* in vulvovaginal Candidiasis. Mycopathologia, 2014, 178 (3, 4): 279–83.

^3^Asadzadeh M, Ahmad S, Hagen F, et al. Simple, Low-Cost Detection of *Candida parapsilosis* complex isolates and molecular fingerprinting of *Candida orthopsilosis* strains in Kuwait by ITS region sequencing and amplified fragment length polymorphism analysis. PLoS One, 2015, 10 (11): e0142880.

^4^Leaw SN, Chang HC, Sun HF, et al. Identification of medically important yeast species by sequence analysis of the internal transcribed spacer regions. J Clin Microb, 2006, 44 (3): 693–9.

### Antifungal susceptibility testing

The in vitro susceptibility tests by using the CLSI reference broth microdilution method were performed for all species isolates number less than 100 strains. *C. albicans* and *C. glabrata* were randomly selected for the test. Those include 1272 *C. albicans* strains (including 998 isolates from VVC and 274 from RVVC) and 267 *C. glabrata* strains(including 197 isolates from VVC and 70 from RVVC). The MIC of Candida for all agents was read following 24–48 h incubation. The antifungals used were amphotericin B (Sigma, USA), Anidulafungin(Selleckchem, USA), Butoconazole(Sigma, USA), Caspofungin(Sigma, USA), Clotrimazole(Sigma, USA), Fluconazole(Sigma, USA), Flucytosine(Sigma, USA), Itraconazole(Sigma, USA), Micafungin(Selleckchem),Miconazole(Sigma, USA), Nystatin (Amresco, USA), Terbinafine(Santa Cruz, USA),Terconazole(Sigma),and Voriconazole(Fluka, USA). Quality control was performed as recommended in CLSI documents M27-A3 and M60 by using ATCC 90028 which is a reference strain of *C. albicans* and all results of the control were within established ranges [20, 21].

### Statistical analysis

All values given in tables and text are expressed as the means unless otherwise indicated. Each variable was tested for differences between groups by Student’s t test or chi-square analysis where appropriate. Statistical significance was set at *P* < 0.05. Statistical analysis of the data was performed using SPSS 10.0 software (SPSS Inc.; Chicago, Illinois, United States).

## Results

### Strain distribution and *y*east identification

**Fig. 1:**
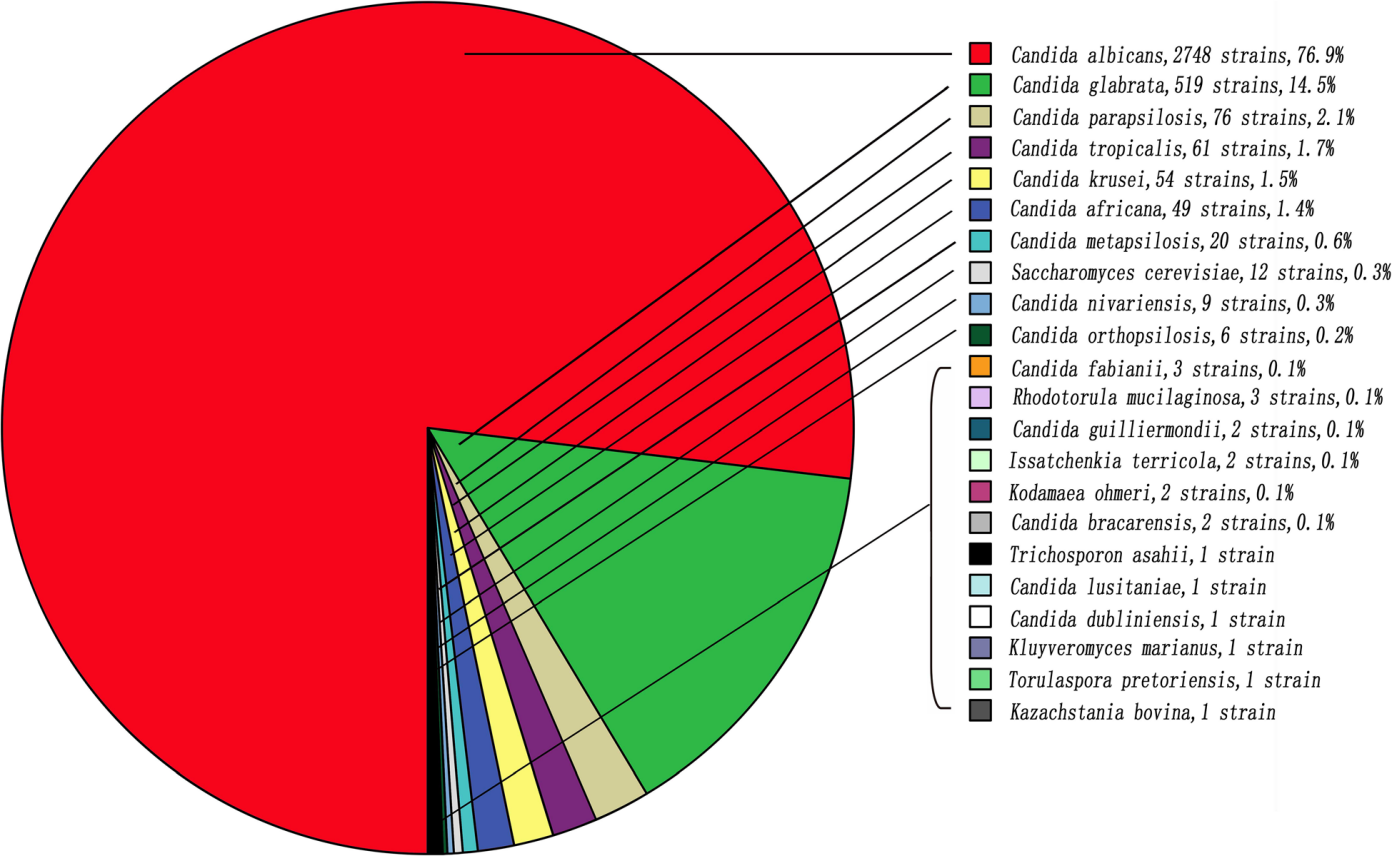
The distribution of the yeast from VVC

**Fig. 2:**
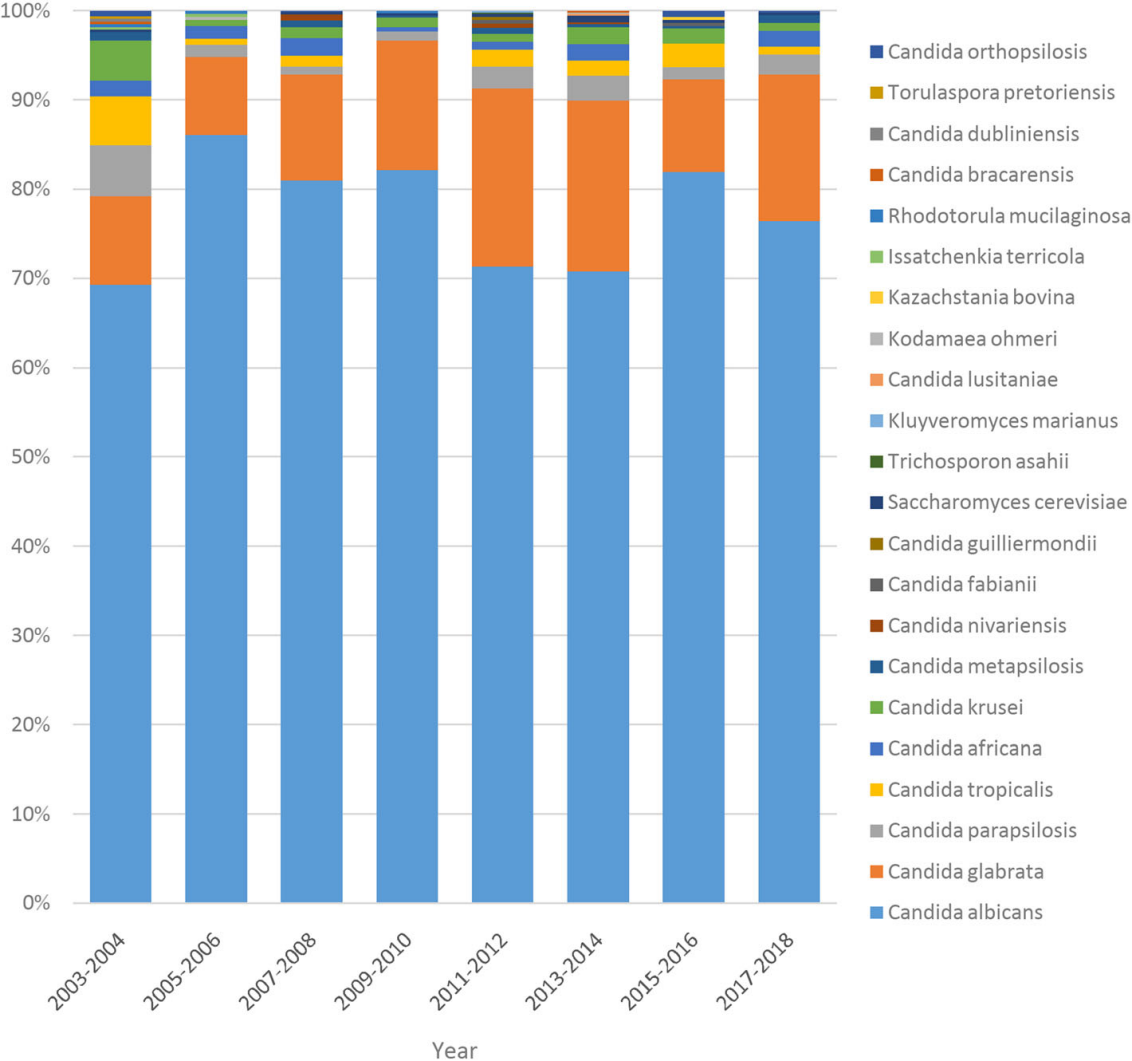
The distribution of the yeast from VVC by years

The 3574 isolates from the vaginal samples represented 22 species based on molecular identification. *C. albicans* were the predominant Candida species (2748 isolates, 76.9%) in VVC, followed by *C. glabrata* (519 isolates, 14.5%), *C. parapsilosis* (76 isolates, 2.1%), and *C. tropicalis* (61 isolates, 1.7%). Fig. 1 shows the distribution of the yeast species from all VVC based on molecular identification**.** Fig. 2 shows the distribution of the yeast species from all VVC by years.

The API 20C system identified only 11 of the species encountered during the study period (Table 2). Among the isolates analysed by the API 20C system, 3273 (91.58%) isolates were correctly identified to the species level. The correct identification rates of *C. albicans, C. glabrata*, *C. parapsilosis,* and *C. tropicalis* were 98.51% (2707/2748 isolates), 84.59% (439/519 isolates), 80.26% (61/76 isolates), and 65.57% (40/61 isolates), respectively*.* The correct identification rate of the API 20C system for rare yeasts, including *C. krusei, Saccharomyces cerevisiae, Candida africana, C. dubliniensis, C. orthopsilosis, C. metapsilosis, C. lusitaniae, C. fabianii, Trichosporon asahii, Rhodotorula, Kodamaea ohmeri,* *C. nivariensis, C. bracarensis, C. guilliermondii, Torulaspora pretoriensis, Kazachstania bovina, Kluyveromyces marianus, and Issatchenkia terricola,* was 15.29% (26/170 isolates) (Table 2**)**.

**Table 2:** Identification of 3574 isolates of yeast from vulvovaginal candidasis based on molecular methods and API 20C

Candida identified by molecular methods (abbreviation)	Candida identified by API 20C (isolates)											Correct identification/total,%
	*CA* *(2866)*	*CG(489)*	CP (85)	CT (57)	CK (27)	SC (24)	*C. famata* (13)	*CL* (4)	*RH* (4)	*C. inconspicua* (3)	*C. humicola* (2)	
*C. albicans (CA)*	2707	24	0	7	4	1	1	0	3	0	1	2707/2748,98.51
*C. africana*	48	1	0	0	0	0	0	0	0	0	0	0/49,0
*C. dubliniensis*	1	0	0	0	0	0	0	0	0	0	0	0/1,0
*C. glabrata (CG)*	52	439	7	7	2	7	4	0	1	0	0	439/519,84.59
*C. nivariensis*	0	7	0	0	1	0	1	0	0	0	0	0/9,0
*C. bracarensis*	0	2	0	0	0	0	0	0	0	0	0	0/2,0
*C. parapsilosis (CP)*	7	4	61	2	0	0	1	1	0	0	0	61/76,80.26
*C. orthopsilosis*	1	1	4	0	0	0	0	0	0	0	0	0/6,0
*C. metapsilosis*	3	5	8	1	0	0	1	1	0	0	1	0/20,0
*C. tropicalis (CT)*	10	2	2	40	0	4	3	0	0	0	0	40/61,65.57
*C. krusei (CK)*	28	2	1	0	18	1	0	1	0	3	0	18/54,33.33
*Saccharomyces cerevisiae (SC)*	2	1	1	0	1	7	0	0	0	0	0	7/12,58.33
*C. lusitaniae (CL)*	0	0	0	0	0	0	0	1	0	0	0	1/1,100
*C. fabianii*	1	0	1	0	0	1	0	0	0	0	0	0/3,0
*Trichosporon asahii ()*	0	0	0	0	0	0	1	0	0	0	0	0/1,0
*Rhodotorula (RH)*	3	0	0	0	0	0	0	0	0	0	0	0/3,0
*C. guilliermondii*	0	0	0	0	0	1	1	0	0	0	0	0/2,0
*Kodamaea ohmeri*	1	0	0	0	0	1	0	0	0	0	0	0/2,0
*Issatchenkia terricola*	0	1	0	0	1	0	0	0	0	0	0	0/2,0
*Torulaspora pretoriensis*	0	0	0	0	0	1	0	0	0	0	0	0/1,0
*Kazachstania bovina*	1	0	0	0	0	0	0	0	0	0	0	0/1,0
*Kluyveromyces marianus*	1	0	0	0	0	0	0	0	0	0	0	0/1,0
*Total*	2866	489	85	57	27	24	13	4	4	3	2	3273/3574,91.58

### Antifungal susceptibility

Antifungal susceptibility was tested in a total of 1844 isolates of Candida from patients with VVC. *C. albicans* was susceptible to most of the tested antifungals including azole, polyenes and echinocandins. The MICs of azoles for *C. glabrata* were higher than those for *C. albicans.* The MICs of echinocandins for *C. parapsilosis* were higher than those for *C. albicans.* Some drug-resistant isolates mainly to azoles were found. *C.albicans* showed 7.7, 10.2 and 6.2% resistance to the fluconazole (MIC ≥8 μg/mL), itraconazole (MIC ≥1 μg/mL) and voriconazole (MIC ≥1 μg/mL), respectively. On the other hand, *C.glabrate* showed 3.4 and 29.1% resistance to the fluconazole (MIC ≥64 μg/mL) and itraconazole (MIC ≥1 μg/mL). In addition, a small number of *C. parapsilosis* were resistant to echinococcins. The resistance rates of *C. parapsilosis* to Anifungin (MIC ≥8 μg/mL), carpofungin (MIC ≥8 μg/mL) and micafungin (MIC ≥8 μg/mL) were 5.2, 5.2 and 1.3%, respectively. The MIC GM value of *C. albicans* for itraconazole, terconazole, and terbinafine in RVVC is higher than those in VVC. The MIC GM value of *C. glabrata* for miconazole, amphotericin B, nystatin, caspofungin, and terbinafine in RVVC is higher than those in VVC (Tables 3 and 4**)**.

**Table 3:** In vitro antifungal susceptibility of 1844 clinical isolates of Candida species as determined by the CLSI method

					Antifungal agents			
Candida species(n)		BUC	CLO	FLC	ITC	MIC	TEC	VRC
*C. albicans, n = 1272*	*Range*	0.015–32	0.015–32	0.06–128	0.015–32	0.015–32	0.015–32	0.015–8
	*GM*	0.11	0.05	0.79	0.09	0.30	0.15	0.07
	*MIC90*	1	0.25	4	1	4	2	0.5
	*R*			7.7%	10.2%			6.2%
*C. africana, n = 49*	*Range*	*0.015–2.*	*0.015–4*	*0.125–1*	*0.015–1*	0.015-0.5	*0.03–16*	*0.03–0.5*
	*GM*	0.04	0.04	0.25	0.04	0.04	0.07	0.04
	*MIC90*	*0.06*	*0.06*	*0.5*	*0.06*	*0.125*	*0.5*	*0.06*
C. *dubliniensis n = 1*	*Range*	0.015	0.015	4	0.125	0.03	0.015	0.015
*C. glabrata, n = 267*	Range	0.015–16	0.015–16	0.125–128	0.015–32	0.015–32	0.015–32	0.015–32
	GM	0.21	0.20	1.48	0.38	0.25	0.16	0.09
	MIC90	1	1	8	4	2	1	0.25
	R			3.4%	29.1%			–
*C. nivariensis, n = 9*	Range	0.03–1	0.03–0.5	0.125–32	0.03–0.5	0.03–16	0.03–1	0.03–4
	*MIC50*	0.03	0.06	2	0.5	0.03	0.03	0.06
	MIC90	0.125	0.125	4	0.5	2	1	0.5
*C. bracarensis, n = 2*	Range	0.03–1	0.03–0.5	0.25–2	0.125–2	0.03–4	0.03–1	0.03–0.125
	*MIC50*	0.03	0.03	0.25	0.125	0.03	0.03	0.03
	MIC90	1	0.5	2	2	4	1	0.125
*C. parapsilosis, n = 76*	Range	0.03–4	0.015–1	0.125–4	0.03–4	0.03–4	0.015–32	0.015–2
	GM	0.18	0.04	0.47	0.07	0.54	0.03	0.04
	MIC90	1	0.06	1	0.25	2	0.03	0.06
	R			0	3%			1%
*C. metapsilosis, n = 20*	Range	0.03–1	0.03–0.25	0.25–4	0.03–1	0.03–8	0.015–0.05	0.015–0.06
	GM	0.16	0.04	0.79	0.08	0.48	0.04	0.03
	MIC90	1	0.06	2	1	4	0.25	0.06
*C. orthopsilosis, n = 6*	Range	0.03–8	0.03–1	0.125–4	0.03–1	0.25–8	0.03–0.25	0.03–0.25
	*MIC50*	0.5	0.06	1.0	0.25	1.0	0.03	0.06
	MIC90	8	1	4	1	8	0.25	0.25
*C. tropicalis, n = 61*	Range	0.03–2	0.015–0.5	0.125–128	0.015–32	0.03–32	0.03–2	0.03–4
	GM	0.15	0.05	0.66	0.07	0.70	0.08	0.05
	MIC90	1	0.25	8	0.125	8	0.25	0.25
	R			10.9%	5.6%			4.3%
*C. krusei, n = 54*	Range	0.06–16	0.015–0.5	0.25–64	0.015–4	0.03–16	0.03–4	0.03–1
	GM	0.85	0.08	15.69	0.27	0.72	0.14	0.21
	MIC90	4	0.5	64	1	8	1	0.5
	R			–	12.5%			0
*Saccharomyces cerevisiae, n = 12*	Range	0.015–2	0.015–1	0.125–32	0.015–2	0.06–8	0.015–0.5	0.03–1
	GM	0.1	0.09	1.2	0.20	0.54	0.11	0.09
	MIC90	0.5	0.5	8	2	4	0.5	0.25
*C. guilliermondii, n = 2*	Range	0.06–4	0.03–4	0.5–16	0.125–16	1–16	0.03–0.25	0.06–1
	*MIC50*	0.06	0.03	0.5	0.125	1	0.03	0.06
	MIC90	4	4	16	16	16	0.25	1
	R			0	50%			0
*C. lusitaniae, n = 1*	Range	0.03	0.03	0.125	0.03	0.03	0.03	0.03
*C.Fabianii, n = 3*	Range	0.03–0.25	0.03–0.06	0.5–1	0.03–0.25	0.125–1	0.03	0.03
	MIC50	0.125	0.03	0.5	0.06	0.125	0.03	0.03
	MIC90	0.25	0.06	1	0.25	1	0.03	0.03
Trichosporon asahii,n = 1	Range	0.03	0.03	0.25	0.03	0.06	0.03	0.03
*Rhodotorula, n = 3*	Range	0.03–0.5	0.03–1	4–128	0.03–8	0.25–8	0.03–0.5	0.03–1
	MIC50	0.06	0.06	64	2	1	0.06	0.03
	MIC90	0.5	1	128	8	8	0.5	1
*Kodamaea ohmeri, n = 2*	Range	0.125–0.5	0.03	0.25–2	0.125–0.25	0.25–0.5	0.03	0.03
	MIC50	0.125	0.03	0.25	0.125	0.25	0.03	0.03
	MIC90	0.5	0.03	2	0.25	0.5	0.03	0.03
*Issatchenkia terricola,n = 2*	Range	1–4	0.06–0.125	32–64	0.25–0.5	0.5	0.25	0.25
	MIC50	1	0.06	32	0.25	0.5	0.25	0.25
	MIC90	4	0.125	64	0.5	0.5	0.25	0.25
*Torulaspora pretoriensis,n = 1*	Range	0.25	0.03	8	0.5	0.5	0.125	0.125
*ATCC90028*^a^	Range	0.015–0.5	0.015–0.5	0.125–2	0.015–4	0.008–0.015	0.015–32	0.015–8
	GM	0.04	0.03	0.21	0.08	0.06	0.03	0.03
	MIC90	0.125	0.03	0.5	0.25	0.015	0.03	0.03
*Candida species(n)*	Antifungal agents							
		AmB	FLU	NYS	TEB	AFG	CFG	MFG
*C. albicans n = 1272*	Range	0.015–32	0.03–128	0.03–32	0.03–256	0.008–0.5	0.008–0.5	0.008–0.5
	GM	0.22	0.70	1.60	45.11	0.015	0.1	0.03
	MIC90	0.5	4	8	256	0.03	0.25	0.25
	R		3.3%			0	0	0
C. africana *n* = 49	Range	0.03–32	0.06–8	0.125–4	0.25–256	0.008–0.03	0.015–0.5	0.008–0.5
	GM	0.08	0.68	0.5	17.31	0.01	0.06	0.02
	MIC90	1	2	4	128	0.015	0.25	0.06
*C. dubliniensis n = 1*	Range	0.06	0.06	0.25	16	0.008	0.015	0.008
*C. glabrata n = 267*	Range	0.03–2	0.06–16	0.03–32	0.25–256	0.008–0.5	0.008–0.5	0.008–0.5
	GM	0.29	0.18	3.39	26.62	0.03	0.11	0.05
	MIC90	1	1	8	256	0.06	0.25	0.25
	R		0			0	0	0
*C. nivariensis n = 9*	Range	0.06–2	0.125–4	0.5–4	1–256	0.015–0.06	0.08–0.5	0.015–0.5
	MIC50	0.06	0.5	1	128	0.06	0.25	0.015
	MIC90	2	2	4	256	0.06	0.5	0.5
*C. bracarensis n = 2*	Range	0.06–1	0.125–2	0.25–8	8–256	0.015–0.03	0.125–0.5	0.015–0.5
	MIC50	0.06	0.125	0.25	8	0.015	0.125	0.015
	MIC90	1	2	8	256	0.03	0.5	0.5
C. parapsilosis *n* = 76	Range	0.03–2	0.125–8	0.03–32	0.25–256	0.008–1	0.008–1	0.008–1
	GM	0.19	0.14	0.59	0.62	0.69	0.60	0.54
	MIC90	1	0.125	4	32	0.5	0.5	0.5
	R		0			5.2%	5.2%	1.3%
C. metapsilosis *n* = 20	Range	0.015–0.5	0.125–4	0.06–4	0.25–256	0.015–0.5	0.008–0.5	0.015–1
	GM	0.10	0.177	0.46	2.17	0.17	0.17	0.39
	MIC90	0.5	1	4	256	0.25	0.25	0.5
*C. orthopsilosis n = 6*	Range	0.06–0.25	0.125–2	0.06–8	0.25–128	0.008–1	0.015–1	0.008–0.5
	MIC50	0.125	1	0.5	64	0.008	0.03	0.25
	MIC90	0.25	2	8	128	1	1	0.5
*C. tropicalis n = 61*	Range	0.03–1	0.125–32	0.03–8	0.25–256	0.015–0.125	0.008–0.5	0.008–0.5
	GM	0.19	0.23	0.54	60.02	0.03	0.24	0.04
	MIC90	0.5	1	4	256	0.06	0.5	0.5
	R		1.8%			0	0	0
*C. krusei n = 54*	Range	0.03–1	0.125–32	0.03–4	16–256	0.015–0.5	0.008–1	0.008–0.5
	GM	0.43	4.2	0.32	75.66	0.08	0.08	0.15
	MIC90	1	16	1	256	0.125	0.5	0.25
	R		2.9%			0	1.85%	0
*Saccharomyces cerevisiae n = 12*	Range	0.03–4	0.06–8	0.125–32	0.25–256	0.015–0.5	0.008–0.5	0.015–0.5
	GM	0.18	0.21	0.78	53.20	0.14	0.10	0.18
	MIC90	1	1	8	256	0.5	0.25	0.25
*C. guilliermondii n = 2*	Range	0.06–0.5	0.125–0.25	0.25–0.5	64–128	0.015–0.25	0.25–0.5	0.015–0.25
	MIC50	0.06	0.125	0.25	64	0.015	0.25	0.015
	MIC90	0.5	0.25	0.5	128	0.25	0.5	0.25
	R		0			0	0	0
*C. lusitaniae n = 1*	Range	0.125	0.125	0.25	0.25	0.03	0.25	0.015
*C. fabianii n = 3*	Range	0.06–0.25	0.125	0.25	128	0.015–0.03	0.015–0.25	0.015–0.06
	MIC50	0.06	0.125	0.25	128	0.015	0.015	0.03
	MIC90	0.25	0.125	0.25	128	0.03	0.25	0.06
*Trichosporon asahii n = 1*	Range	0.125	1	0.25	128	0.015	0.5	0.015
*Rhodotorula n = 3*	Range	0.03–0.5	0.125	0.06–0.125	8–256	0.06–0.5	0.25–0.5	0.25–0.5
	MIC50	0.03	0.125	0.06	8	0.06	0.25	0.25
	MIC90	0.5	0.125	0.125	256	0.5	0.5	0.5
*Kodamaea ohmeri n = 2*	Range	0.125–1	0.125	0.5	128	0.015	0.125–0.25	0.015–0.03
	MIC50	0.125	0.125	0.5	128	0.015	0.125	0.015
	MIC90	1	0.125	0.5	128	0.015	0.25	0.03
*Issatchenkia terricola n = 2*	Range	0.125–0.5	8	0.5	128–256	0.06	0.06	0.008
	MIC50	0.125	8	0.5	128	0.06	0.06	0.008
	MIC90	0.5	8	0.5	256	0.06	0.06	0.008
*Torulaspora pretoriensis n = 1*	Range	0.125	0.125	0.25	16	0.015	0.125	0.015
*ATCC90028*^a^	Range	0.03–2	0.125–8	0.25–16	1–256	0.008–0.015	0.015–0.5	0.008–0.015
	GM	0.22	0.64	1.31	88.22	0.01	0.09	0.01
	MIC90	1	2	8	256	0.015	0.5	0.015

Note: *GM* geometry mean, *BUC* butoconazole, *CLO* Clotrimazole, *FLC* Fluconazole, *ITC* Itraconazole, *VRC* Voriconazole, *MIC* Miconazole, *TEC* Terconazole, *AmB* Amphotericin B, *FLU* Flucytosine, *NYS* Nystatin, *TEB* Terbinafine, *AFG* Anidulafungin, *CFG* Caspofungin, *MFG* Micafungin

^a^ *ATCC90028 was tested 57 times*

**Table 4:** In vitro antifungal susceptibility of 1539 clinical *C.* albicans and *C. glabrata* isolates from VVC and RVVC as determined by the CLSI method

		AmB	FLU	NYS	TEB	AFG	CFG	MFG	BUC	CLO	FLC	ITC	MIC	TEC	VRC
*C. albicans*															
VVC	Range	0.15–32	0.030–128	0.03–32	0.03–256	0.008–0.5	0.008–0.5	0.008–0.5	0.015–32	0.015–32	0.06–128	0.015–32	0.015–32	0.015–32	0.015–8
	GM	0.21	0.65	1.55	39.23	0.02	0.1	0.03	0.11	0.05	0.74	0.09	0.27	0.12	0.06
	MIC90	0.5	4	8	256	0.03	0.25	0.25	1	0.25	4	1	4	2	0.5
RVVC	Range	0.015–32	0.06–128	0.03–32	0.25–256	0.008–0.25	0.008–0.5	0.008–0.5	0.015–8	0.015–16	0.06–64	0.015–32	0.015–32	0.015–32	0.015–8
	GM	0.25	0.94	1.82	74.50	0.01	0.1	0.03	0.13	0.05	1.03	0.10	0.46	0.33	0.09
	MIC90	0.6	4	8	256	0.015	0.25	0.06	1	0.5	4	0.5	8	8	0.5
	P	0.813	0.435	0.770	0	0.242	0.7	0.566	0.736	0.341	0.14	0.041	0.053	0	0.34
*C. glabrata*															
VVC	Range	0.03–2	0.06–16	0.03–32	0.25–256	0.015–0.5	0.008–0.5	0.008–0.5	0.015–16	0.015–16	0.125–128	0.015–32	0.015–16	0.015–32	0.015–32
	GM	0.33	0.18	3.88	40.63	0.03	0.06	0.05	0.24	0.23	1.68	0.42	0.25	0.15	0.09
	MIC90	1	1	8	256	0.06	0.25	0.25	1	1	8	4	2	1	0.25
RVVC	Range	0.03–2	0.06–2	0.03–16	0.25–256	0.008–0.06	0.008–0.5	0.008–0.5	0.015–8	0.03–4	0.125–128	0.03–32	0.03–32	0.015–32	0.015–32
	GM	0.20	0.17	2.31	8.86	0.02	0.06	0.04	0.16	0.134	1.03	0.29	0.27	0.17	0.11
	MIC90	0.5	1	8	256	0.03	0.25	0.25	1	1	16	2	4	2	0.5
	P	0.017	0.366	0.035	0.015	0.058	0.68	0.78	0.87	0.25	0.1	1	0.043	0.683	0.293

Note: *GM* geometry mean, *BUC* butoconazole, *CLO* Clotrimazole, *FLC* Fluconazole, *ITC* Itraconazole, *VRC* Voriconazole, *MIC* Miconazole, *TEC* Terconazole, *AmB* Amphotericin B, *FLU* Flucytosine, *NYS* Nystatin, *TEB* Terbinafine, *AFG* Anidulafungin, *CFG* Caspofungin, *MFG* Micafungin. The MIC GM value of *C. albicans* for Itraconazole, Terconazole, and Terbinafine in RVVC is higher than those in VVC. The MIC GM value of *C. glabrata* for Miconazole, Amphotericin B, Nystatin, Caspofungin, and Terbinafine in RVVC is higher than those in VVC

## Discussion

### Strain identification and distribution

Borman reported 1781 yeast isolates submitted to the United Kingdom Mycology Reference Laboratory and found that 100 isolates (9.7%) were incorrectly identified, with error rates ranging from 5.2 to 18.2% [22]. The conventional methods such as the API ID 32 C system could not identify the rare or new recovered Candida [23]. The identification ratios (IR) at the species level of yeast were 0.89 for the API ID 32C system, 0.89 for the AuxaColor system, and 0.93 for the Vitek 2 system. Subanalysis of data showed that the Vitek 2 system was more accurate (IR: 0.94) than the API ID32C system (IR: 0.84) and the AuxaColor system (IR: 0.76) [7]. Gündeş reported the performance of API 20C Aux was with 87% (101 of 116 isolates) [24]. Two hundred and fifty-one isolates (83.7%) were correctly identified, 49 (16.2%) isolates were misidentified, and there was no species without identification using API 20C AUX. The majority of misidentified yeast isolates were among rare species (*n* = 45), and the majority (4/5) of *Pichia kudriavzevii* strains were misidentified [25]. The closely related Candida complex was identified from vaginal samples by using molecular methods [8, 16–19, 26]. Based on conventional and molecular methods, *C. albicans*, *C. glabrata*, *C. parapsilosis*, and *C. tropicalis* are the four most common Candida species from VVC. Most of previous studies were non-molecular identification or small samples based molecular identification [3–6, 27, 28]. In the current study, by using molecular identification, we found that *C. albicans* was still the most common Candida species in VVC, followed by *C. glabrata*, *C. parapsilosis* and *C. tropicalis.* The yeast species from VVC was stable in the past 16 years.

The API 20C system has a lower correct identification rate for non-albicans (33.33–84.59%) than that for *C. albicans* (98.51%). The system also could not identify new closely related Candida species and novel species. Compared with conventional methods by which 5–10 Candida species were identified, molecular methods identified more than 20 Candida species from vaginal samples, suggesting the necessity of molecular identification in research [3–5, 22].

### Antifungal susceptibility

Most non-albicans Candida species have a higher azole MICs, and the VVC they cause are often difficult to treat [28–35]. Fluconazole-resistant *C. albicans* have been found in VVC [34, 35].

The antifungal prescription affects the relative distribution and susceptibility of Candida [36, 37].

An increasing number of isolates with elevated MICs were observed following fluconazole introduction rather than prior to that [37]. In our current study, *C. albicans* was susceptible to most of the tested antifungals. The MICs of azoles for *C. glabrata* were higher than those for *C. albicans* and the MICs of echinocandins for *C. parapsilosis* were higher than those for *C. albicans,* which were similar to a previous study [32]. The MICs of nystatin for *C. albicans and C. glabrata* were higher than that from the findings of other reports and those of our previous study on the use of different antifungal susceptibility tests [3, 38]. In current study, terbinafine was the less active drug against most of the tested isolates, which was similar to a previous study and may not be used for treating VVC [24, 39]. CD101, a new echinocandin antifungal agent, has been studied specifically as a possible treatment for VVC in rat and human [12–15]. The current study has shown that echinocandins including anidulafungin, caspofungin and micafungin have a low MIC for *C. glabrata,* which may provide an opportunity for treating azole-resistant VVC.

## Conclusions

It was concluted that API 20C yeast identification system can be used to reliably identify the most common Candida species. Molecular methods are necessary for the identification of closely related, emerging, and rare yeast species, which are quite important in research. *C. albicans* was the predominant Candida species isolated from this sample of patients with VVC. The results from this study suggest that much of the previous studies of epidemiology for VVC should be re-thought. Resistance of vaginal *C. albicans* isolates to antifungal agents was infrequent.

## Data Availability

The datasets used during the current study are available from the corresponding author on reasonable request.

## Abbreviations

CLSI: Clinical & Laboratory Standards Institute
GM: Geometric mean
MIC: Minimal inhibitory concentration
PCR: Polymerase chain reaction
VVC: Vulvovaginal candidiasis
RVVC: Recurrent VVC
SPSS: Statistical product and service solutions
