# Correction to: Molecular identification and antifungal susceptibility profile of yeast from vulvovaginal candidiasis

**DOI:** 10.1186/s12879-021-06601-x

**Published:** 2022-01-24

**Authors:** Yu Shi, Yuxia Zhu, Shangrong Fan, Xiaoping Liu, Yiheng Liang, Yingying Shan

**Affiliations:** 1 Department of Obstetrics and Gynecology, Peking University Shenzhen Hospital, Shenzhen, 518036 China; 2 Shenzhen Key Laboratory of Gynecological Diagnostic Technology Research, Shenzhen, 518036 China; 3 Anhui Medical University, Hefei, 230022 China; 4 Department of Laboratory Science, Peking University Shenzhen Hospital, Shenzhen, 518036 China

## Correction to: BMC Infectious Diseases (2020) 20:287 10.1186/s12879-020-04985-w

Following publication of the original article [1], the authors identified an error in Table 3. The correct table (Table 3) is given in this erratum and the original article has been corrected.

**Table 3:** In vitro antifungal susceptibility of 1844 clinical isolates of Candida species as determined by the CLSI method

*Candid*a species (n)	Antifungal agents							
		BUC	CLO	FLC	ITC	MIC	TEC	VRC
*C. lusitaniae, n* = *1*	Range	0.03	0.03	0.125	0.03	0.03	0.03	0.03
*C. Fabianii, n* = *3*	Range	0.03–0.25	0.03–0.06	0.5–1	0.03–0.25	0.125–1	0.03	0.03
	MIC50	0.125	0.03	0.5	0.06	0.125	0.03	0.03
	MIC90	0.25	0.06	1	0.25	1	0.03	0.03
Trichosporon asahii, n = 1	Range	0.03	0.03	0.25	0.03	0.06	0.03	0.03
*Rhodotorula, n* = *3*	Range	0.03–0.5	0.03–1	4–128	0.03–8	0.25–8	0.03–0.5	0.03–1
	MIC50	0.06	0.06	64	2	1	0.06	0.03
	MIC90	0.5	1	128	8	8	0.5	1
*Kodamaea ohmeri, n* = *2*	Range	0.125–0.5	0.03	0.25–2	0.125–0.25	0.25–0.5	0.03	0.03
	MIC50	0.125	0.03	0.25	0.125	0.25	0.03	0.03
	MIC90	0.5	0.03	2	0.25	0.5	0.03	0.03
*Issatchenkia terricola, n* = *2*	Range	1–4	0.06–0.125	32–64	0.25–0.5	0.5	0.25	0.25
	MIC50	1	0.06	32	0.25	0.5	0.25	0.25
	MIC90	4	0.125	64	0.5	0.5	0.25	0.25
*Torulaspora pretoriensis, n* = *1*	Range	0.25	0.03	8	0.5	0.5	0.125	0.125
ATCC90028*	Range	0.015–0.5	0.015–0.5	0.125–2	0.015–4	0.008–0.015	0.015–32	0.015–8
	GM	0.04	0.03	0.21	0.08	0.06	0.03	0.03
	MIC90	0.125	0.03	0.5	0.25	0.015	0.03	0.03

*Candida* species (n)	Antifungal agents							
		AmB	FLU	NYS	TEB	AFG	CFG	MFG
*C. albicans* *n* = *1272*	Range	0.015–32	0.03–128	0.03–32	0.03–256	0.008–0.5	0.008–0.5	0.008–0.5
	GM	0.22	0.70	1.60	45.11	0.015	0.1	0.03
	MIC90	0.5	4	8	256	0.03	0.25	0.25
	R		3.3%			0	0	0
*C. africana* n = 49	Range	0.03–32	0.06–8	0.125–4	0.25–256	0.008–0.03	0.015–0.5	0.008–0.5
	GM	0.08	0.68	0.5	17.31	0.01	0.06	0.02
	MIC90	1	2	4	128	0.015	0.25	0.06
*C. dubliniensis* *n* = *1*	Range	0.06	0.06	0.25	16	0.008	0.015	0.008
*C. glabrata* *n* = *267*	Range	0.03–2	0.06–16	0.03–32	0.25–256	0.008–0.5	0.008–0.5	0.008–0.5
	GM	0.29	0.18	3.39	26.62	0.03	0.11	0.05
	MIC90	1	1	8	256	0.06	0.25	0.25
	R		0			0	0	0
*C. nivariensis* *n* = *9*	Range	0.06–2	0.125–4	0.5–4	1–256	0.015–0.06	0.08–0.5	0.015–0.5
	MIC50	0.06	0.5	1	128	0.06	0.25	0.015
	MIC90	2	2	4	256	0.06	0.5	0.5
*C. bracarensis* *n* = *2*	Range	0.06–1	0.125–2	0.25–8	8–256	0.015–0.03	0.125–0.5	0.015–0.5
	MIC50	0.06	0.125	0.25	8	0.015	0.125	0.015
	MIC90	1	2	8	256	0.03	0.5	0.5
*C. parapsilosis* n = 76	Range	0.03–2	0.125–8	0.03–32	0.25–256	0.008–1	0.008–1	0.008–1
	GM	0.19	0.14	0.59	0.62	0.69	0.60	0.54
	MIC90	1	0.125	4	32	0.5	0.5	0.5
	R		0			5.2%	5.2%	1.3%
*C. metapsilosis* n = 20	Range	0.015–0.5	0.125–4	0.06–4	0.25–256	0.015–0.5	0.008–0.5	0.015–1
	GM	0.10	0.177	0.46	2.17	0.17	0.17	0.39
	MIC90	0.5	1	4	256	0.25	0.25	0.5

*Candida* species (n)	Antifungal agents							
		AmB	FLU	NYS	TEB	AFG	CFG	MFG
*C. orthopsilosis* *n* = *6*	Range	0.06–0.25	0.125–2	0.06–8	0.25–128	0.008–1	0.015–1	0.008–0.5
	MIC50	0.125	1	0.5	64	0.08	0.30	0.25
	MIC90	0.25	2	8	128	1	1	0.5
*C. tropicalis* *n* = *61*	Range	0.03–1	0.125–32	0.03–8	0.25–256	0.015–0.125	0.008–0.5	0.008–0.5
	GM	0.19	0.23	0.54	60.02	0.03	0.24	0.04
	MIC90	0.5	1	4	256	0.06	0.5	0.5
	R		1.8%			0	0	0
*C. krusei* *n* = *54*	Range	0.03–1	0.125–32	0.03–4	16–256	0.015–0.5	0.008–1	0.008–0.5
	GM	0.43	4.2	0.32	75.66	0.08	0.08	0.15
	MIC90	1	16	1	256	0.125	0.5	0.25
	R		2.9%			0	1.85%	0
*Saccharomyces cerevisiae* *n* = *12*	Range	0.03–4	0.06–8	0.125–32	0.25–256	0.015–0.5	0.08–0.5	0.015–0.5
	GM	0.18	0.21	0.78	53.20	0.14	0.10	0.18
	MIC90	1	1	8	256	0.5	0.25	0.25
*C. guilliermondii* *n* = *2*	Range	0.06–0.5	0.125–0.25	0.25–0.5	64–128	0.015–0.25	0.25–0.5	0.015–0.25
	MIC50	0.06	0.125	0.25	64	0.015	0.25	0.015
	MIC90	0.5	0.25	0.5	128	0.25	0.5	0.25
	R		0			0	0	0
*C. lusitaniae* *n* = *1*	Range	0.125	0.125	0.25	0.25	0.03	0.25	0.015
*C. fabianii* *n* = *3*	Range	0.06–0.25	0.125	0.25	128	0.015–0.03	0.015–0.25	0.015–0.06
	MIC50	0.06	0.125	0.25	128	0.015	0.015	0.03
	MIC90	0.25	0.125	0.25	128	0.03	0.25	0.06
*Trichosporon asahii* *n* = *1*	Range	0.125	1	0.25	128	0.015	0.5	0.015
*Rhodotorula* *n* = *3*	Range	0.03–0.5	0.125	0.06–0.125	8–256	0.06–0.5	0.25–0.5	0.25–0.5
	MIC50	0.03	0.125	0.06	8	0.06	0.25	0.25
	MIC90	0.5	0.125	0.125	256	0.5	0.5	0.5
*Kodamaea ohmeri* *n* = *2*	Range	0.125–1	0.125	0.5	128	0.015	0.125–0.25	0.015–0.03
	MIC50	0.125	0.125	0.5	128	0.015	0.125	0.015
	MIC90	1	0.125	0.5	128	0.015	0.25	0.03
*Issatchenkia terricola* *n* = *2*	Range	0.125–0.5	8	0.5	128–256	0.06	0.06	0.08
	MIC50	0.125	8	0.5	128	0.06	0.06	0.08
	MIC90	0.5	8	0.5	256	0.06	0.06	0.08
*Torulaspora pretoriensis* *n* = *1*	Range	0.125	0.125	0.25	16	0.015	0.125	0.015
*ATCC90028*^a^	Range	0.03–2	0.125–8	0.25–16	1–256	0.008–0.015	0.015–0.5	0.008–0.015
	GM	0.22	0.64	1.31	88.22	0.01	0.09	0.01
	MIC90	1	2	8	256	0.015	0.5	0.015

*GM* geometry mean, *BUC* butoconazole, *CLO* Clotrimazole, *FLC* Fluconazole, *ITC* Itraconazole, *VRC* Voriconazole, *MIC* Miconazole, *TEC* Terconazole, *AmB* Amphotericin B, *FLU* Flucytosine, *NYS* Nystatin, *TEB* Terbinafine, *AFG* Anidulafungin, *CFG* Caspofungin, *MFG* Micafungin

^a^ATCC90028 was tested 57 times
